# Pharmacokinetic interactions between three SGLT2 inhibitors and telmisartan: A focus on empagliflozin, ertugliflozin, and henagliflozin

**DOI:** 10.1371/journal.pone.0350400

**Published:** 2026-06-22

**Authors:** Xin Zhou, Yanru Deng, Caihui Guo, Ying Li, Yuhao Fu, Zhi Wang, Zhanjun Dong

**Affiliations:** 1 Graduate School, Hebei Medical University, Shijiazhuang, China; 2 Hebei General Hospital, Hebei Key Laboratory of Clinical Pharmacy, Shijiazhuang, China; 3 Department of Pharmacy, Hebei Chest Hospital, Shijiazhuang, China; Kohat University of Science and Technology (KUST), PAKISTAN

## Abstract

**Background:**

Patients with type 2 diabetes mellitus (T2DM) usually accompany with the occurrence of high blood pressure, necessitating a combination of treatments. This includes sodium-glucose co-transporter 2(SGLT2) inhibitors like empagliflozin, ertugliflozin, and henagliflozin, and antihypertensives like telmisartan. Both classes interact with transporters like P-glycoprotein (P-gp) and breast cancer resistance protein (BCRP), with telmisartan further inhibiting several, including organic anion transporting poly-peptide (OATP) 1B1/1B3.

**Objective:**

Despite their common use, pharmacokinetic interactions between these drugs remain underexplored. This study aims to investigate the potential pharmacokinetic interaction between three specific SGLT2 inhibitors and telmisartan.

**Methods:**

Rats were divided into twelve groups, with six rats per group, and received different combinations of empagliflozin, ertugliflozin, henagliflozin, and telmisartan. Drug concentrations were measured using ultra-performance liquid chromatography-tandem mass spectrometry, and mRNA expressions through quantitative reverse transcription polymerase chain reaction (RT-qPCR).

**Results:**

Our study manifested that telmisartan increased the plasma concentration-time curves (AUC_0-t_ and AUC_0–∞_) and the maximum plasma concentrations (C_max_) of empagliflozin, whereas the apparent clearance (CL_z/F_) and apparent volume of distribution (V_z_) significantly decreased(all *p* ＜ 0.05). Similarly, telmisartan increased the AUC_0-t_, AUC_0-∞_ and C_max_ of henagliflozin and decreased the CL_z/F_(all *p* ＜ 0.05). When coadministered with ertugliflozin or henagliflozin, the AUC_0-t_ and AUC_0–∞_ of telmisartan decreased significantly and the CL_z/F_ increased significantly(all *p* ＜ 0.05). Furthermore, PCR results demonstrated that telmisartan decreased the expression of BCRP expression in liver, intestines and kidney, P-gp expression in the intestines and kidney and OATP1B2 expression in liver tissue.

**Conclusions:**

Our findings highlight the importance of these drug interactions, which could inform dose adjustments to enhance safety in patients with T2DM and hypertension.

## Introduction

Diabetes Mellitus is a multifactorial chronic health condition influenced by a combination of genetic and environmental factors [1, 2]. In 2021, an estimated 537 million people were living with diabetes, a figure expected to surge by 46% to 783 million by 2045 [3]. SGLT2 inhibitors represent a first-line antidiabetic medicines and have found broad applications in heart and kidney protection. They offer a range of pharmacological benefits, including lowering blood glucose, enhancing insulin sensitivity, and safeguarding the heart and kidneys. SGLT2 is a protein transporter primarily localized in the proximal renal tubules, where its main role is to reabsorb the majority of glucose filtered by the kidneys. By inhibiting SGLT2, these medicines facilitate urinary excretion of excess glucose so as to reduce blood sugar levels [4]. Additionally, SGLT2 inhibitors have demonstrated lighten in weight and blood pressure [4].

**Fig 1:**
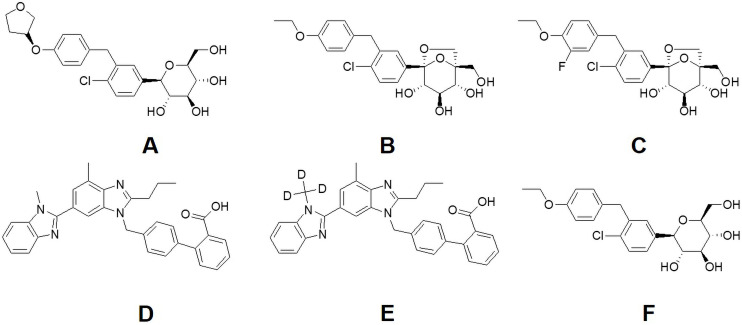
Molecular structures of empagliflozin (A), ertugliflozin (B), henagliflozin (C), telmisartan (D), telmisartan-d_3_ (E) and dapagliflozin (F).

Empagliflozin, ertugliflozin and henagliflozin (Fig 1) are three relatively new SGLT2 inhibitors. The selectivity of empagliflozin was the strongest, followed by ertugliflozin, and the weakest to henagliflozin [5,6]. The curative effect of three medicines has been confirmed in clinical trials and showed additional curative effects like alleviate the risk of adverse cardiovascular events [7–9]. All three drugs, which are SGLT2 inhibitors, differ only slightly in structure and therefore exhibited different pharmacokinetics characteristics like the plasma protein binding rate, the time to maximum concentration (T_max_), uridine diphosphate-glucuronosyltransferase (UGT) metabolic enzyme system, the half-life(t_1/2_), and in vivo clearance pathway. The plasma protein binding rate of henagliflozin (94.5–95.9%) was slightly higher than that of ertugliflozin (93.6%) and higher than that of empagliflozin (86.2%) [10–12]. The metabolism of empagliflozin, ertugliflozin, and henagliflozin involves different UGT enzymes: UGT2B7, UGT1A3, UGT1A8, and UGT1A9 for empagliflozin; UGT1A9 and UGT2B7 for ertugliflozin [13,14]; and UGT2B4/7, UGT1A9, UGT1A3, and UGT1A6 for henagliflozin [12]. Empagliflozin is a substrate for the uptake transporters OATP1B1 and OATP1B3 [15]. All three SGLT2 inhibitors are substrates of P-gp and BCRP [10,11]. From the above, we can conclude that SGLT2 inhibitors may have potential drug interactions with other medications and have limited information available regarding interactions with other medications at present.

Hypertension presents an immediate and critical concern, closely correlated with elevated risks of cardiovascular morbidity and mortality. Over two-thirds of the sick with T2DM exhibit high blood pressure, and the advancement of hypertension tends to coincide with the progression of hyperglycemia [16]. Previous observational studies have indicated that hyperinsulinemia and insulin resistance may also play a significant role in patients with hypertension [17]. These conditions induced vascular remodeling through the activation of the sympathetic nervous system and the renin-angiotensin-aldosterone system, consequently leading to elevated peripheral vascular resistance, increased cardiac output, and expanded circulating fluid volume [18].Telmisartan, commonly prescribed for hypertension treatment, is a highly selective Angiotensin II type 1 receptor blocker (ARB). Clinical studies have shown that telmisartan moderately decreases multiple adverse cardiovascular events risk [19]. Additionally, telmisartan showing value in modulating glucose and lipid metabolism as well as improving insulin resistance [20]. Telmisartan was metabolized by UGT1A3 in very small amounts, and the majority of its effects were exerted in its original form [21]. In vitro studies have identified telmisartan as a substrate and inhibitor of efflux transporters like P-gp, BCRP, and MRP2 [22,23]. Recent research has indicated that telmisartan may have drug–drug interactions (DDIs) with multiple type of drug like digoxin, rosuvastatin and lenvatinib [24–26]. Therefore, the risk of DDIs between telmisartan and other medications was very high.

In clinical practice, ARBs and antidiabetic agents are often prescribed for extended durations, thereby heightening the potential for DDIs. Despite this, there exists a lack of comprehensive studies analyzing the DDIs between telmisartan and specific SGLT2 inhibitors like empagliflozin, ertugliflozin, and henagliflozin. To bridge this knowledge gap, the current study was designed to assess the pharmacokinetic interactions between telmisartan and these antidiabetic drugs in rats, employing a verified ultra-performance liquid chromatography-tandem mass spectrometry (UPLC-MS/MS) methodology. Moreover, we explored the changes of telmisartan in the mRNA levels of efflux transporters P-gp and BCRP in the liver, intestinal, and renal tissues of rats and to inquiry the underlying mechanisms that may contribute to these interactions.

## Materials and methods

### Animals

A total of 72 healthy male Sprague–Dawley rats (250 ± 20g), were purchased from Beijing Huafukang Biotechnology Co., Ltd. (Beijing, China; license number: SCXK [Jing] 2019−0008). The research protocol was endorsed by the Animal Ethics Committee of Hebei General Hospital (Shijiazhuang, China) under approval number No. 2023149. The welfare of the laboratory animals followed the Guidelines for the Ethical Review of Laboratory Animal Welfare People’s Republic of China National Standard GB/T 35892–20181. Before experimentation, the animals underwent a seven-day acclimation cycle under standard laboratory circumstances: a 12-hour light/dark cycle, a controlled temperature of approximately 23 ± 2 °C, and a relative humidity of 50 ± 10%. Eating and drinking were getatable optionally ad libitum, except eating was forbid 12 hours before drug administration.

### Materials

Empagliflozin (J19HS174525, ≥ 98%) and telmisartan (Y20A7C13363, ≥ 98%) were purchased from Shanghai Yuan ye Biotechnology Co. Ltd. (Shanghai, China). Ertugliflozin was obtained from Shanghai Macklin Biochemical Technology Co., Ltd. (Shanghai, China). Henagliflozin (purity ≥99%) was kindly provided by the Jiangsu Heng Rui Medicine Co., Ltd. (Jiangsu, China). The internal standard (IS), dapagliflozin (f2209347, purity ≥ 99%) was from Shanghai Aladdin Biochemical Technology Co., Ltd. (Shanghai, China). Telmisartan-d_3_ was purchased from TLC Pharmaceutical Standards (Aurora, ON, Canada). For the analytical procedures, Acetonitrile, formic acid, ammonium acetate, and ethyl acetate of high-performance liquid chromatography (HPLC) grade were supplied by Fisher Scientific (Pittsburgh, PA, USA). Ultrapure water was gained from Wahaha Group Co., Ltd. (Hangzhou, China). Additionally, key reagents for RNA extraction and quantitative reverse transcription polymerase chain reaction (RT-qPCR) such as the TRNzol Universal Reagent, FastKing RT Kit (with DNase), and SuperReal PreMix Plus (SYBR Green) were provided by Tiangen Biotechology Co., Ltd. (Beijing, China).

### Pharmacokinetic study in rats

The experimental animals were randomly divided into 12 drug treatment groups (n = 6). The sample size for each group of rats was calculated using the resource equation method [27]. The doses were chosen by converting the clinically recommended doses for patients to animal doses. The animal equivalent dose was determined by dose-by-factor method: Animal equivalent dose (mg / kg) = Human does (mg / kg) × Km ratio (Km = 6.2 in rats) [28]. The value of Km represents the equivalent dose ratio between humans and rats, calculated based on body surface area. This experiment followed the ARRIVE(Animal Research: Reporting of In Vivo Experiments) reporting guidelines [29]. The drugs empagliflozin, ertugliflozin, henagliflozin, and telmisartan were suspended in 0.5% sodium carboxymethyl cellulose (CMC-Na). The following groups received control solvents corresponding to each drug for a duration of seven consecutive days. A seven day treatment period was selected based on previous pharmacokinetic interaction studies employing a similar design [30].On the seventh day, these groups were administered empagliflozin at 2.5 mg/kg (Group 3), ertugliflozin at 1.5 mg/kg (Group 7), henagliflozin at 1 mg/kg (Group 11), or telmisartan at 8 mg/kg (Groups 1, 5, and 9) via oral gavage. Conversely, remaining groups were treated with their respective drugs at the aforementioned doses for seven consecutive days. On the seventh day, these groups adopted a second dose of telmisartan at 8 mg/kg (Groups 4, 8, and 12), empagliflozin at 2.5 mg/kg (Group 2), ertugliflozin at 1.5 mg/kg (Group 6), or henagliflozin at 1 mg/kg (Group 10), also via oral gavage.

100 μL of the blood was collected into heparinized test tubes at the following time points: 0, 0.083, 0.25, 0.5, 1, 1.5, 2, 3, 4, 5, 6, 8, 10, 12, 24, 48, and 72 h for telmisartan; 0, 0.083, 0.167, 0.25, 0.333, 0.5, 0.75, 1, 1.5, 2, 3, 4, 6, 8, 10, 12, 24, and 48h for ertugliflozin analysis. The blood sampling times for empagliflozin and henagliflozin were based on the previous article by our research group [31]. Blood samples were centrifuged at 3,500 rpm for 10 min, and the supernatant was collected and stored at −80℃ until processing for UPLC-MS/MS.

The induction of anesthesia was performed using 5% isoflurane, and the concentration was maintained at 2% throughout the blood collection process. The blood was extracted from the eye venous plexus by well-trained professionals to minimize the animal’s suffering. Following blood collection, rat liver, intestinal and kidney tissues were collected for molecular analysis on the seventh day post-treatment with the corresponding drugs for each group. Before euthanizing the animals, they were required to fast for at least 12 hours. Using sodium pentobarbital (2 g/kg) to put the animals into a deep state of anesthesia, Collect the required tissue samples and the rats were euthanized by cervical dislocation. Then, these animals were under deep anesthesia during the tissue sample collection process and had not regained consciousness yet, so no additional analgesic drugs were necessary.

### Instruments and analytical conditions

Due to the fact that the drugs used in this experiment were different from those in the previous experiments conducted by our research group, improvements were made based on the original experimental methods, and methodological validation was also carried out [31]. The temperature maintained at 40 by gradient elution. The flow rate was 0.3 mL/min and the gradient elution procedure was as follows: 0–0.5 min, 50–60% B; 0.5–2.7 min, 60% B; 2.7–2.8 min, 60–90% B; 2.8–3.8 min, 90% B; 3.8–3.9 min, 90–50% B; 3.9–4.9 min, 90% B. The injection volume was 8 μL. The mass spectrometry conditions for other drugs are as follows: 437.1 → 207.0 for ertugliflozin, 515.3 → 497.2 for telmisartan, 518.4 → 279.1 for telmisartan-d_3_ and 426.4 → 167.1 for dapagliflozin, respectively. The conditions of the mass spectrometer were given as follows: ion source gas 1, 60.0 psi; ion source gas 2, 50.0 psi; curtain gas, 25.0 psi; source temperature, 500；ion spray voltage, 5500 V.

### Preparation of calibration standards and quality control samples

The preparation methods for calibrating standard substances and quality control (QC) samples are referred to the published articles of our research group [31]. The following final concentration ranges for the calibration curves: TEL (2, 10, 50, 75, 100, 150, 300, and 500 ng/mL) and empagliflozin, ertugliflozin, and henagliflozin (5, 20, 50, 200, 500, 1,000, and 2,000 ng/mL). Low-, medium-, and high-concentration QC samples were prepared separately, containing telmisartan concentrations of 5, 200, and 400 ng/mL; and empagliflozin, ertugliflozin, and henagliflozin concentrations of 10, 800, and 1,500 ng/mL, respectively.

### Plasma sample preparation

In this experiment, we improved the analytical methods previously published by our research group related to this topic. 50 µL of plasma sample added up with 5 µL of the IS working solution (either dapagliflozin or telmisartan-d_3_), mixed by the addition of 250 µL of ethyl acetate, then thoroughly vortexed for three minutes and centrifuged in 12,000 rpm for 10 minutes. A 150 µL liquid supernatant was gathered and evaporated to dryness under a stream of nitrogen gas. The residues of empagliflozin, ertugliflozin, henagliflozin, and telmisartan were redissolved in 100 µL of a 50% acetonitrile water compound. Aliquots (8 µL) of these redissolved samples were used into the UPLC-MS/MS system for subsequent analysis.

### Method validation

The UPLC-MS/MS method was strictly verified in line with the Bioanalytical Method Validation Guidance for Industry as stipulated by the United States Food and Drug Administration(US FDA, 2022) [32]. Method validation parameters include specificity, calibration, lower limit of quantification (LLOQ), accuracy, precision, matrix effects, recovery, and stability.

#### Selectivity.

Specificity was assessed by analyzing the chromatograms of blank plasma samples originating from six different rats, either untreated or spiked with IS and the target analytes at the LLOQ level. Additional comparisons were made with actual rat plasma samples post-administration of empagliflozin, ertugliflozin, henagliflozin or telmisartan. The peak area of the blank plasma at the analyte retention time should not more than 20% of the LLOQ and 5% of the IS in order to eliminate the likelihood of obstruct from endogenous substances.

#### Linearity and LLOQ.

Calibration curves were evaluated at empagliflozin (5–2,000 ng/mL), ertugliflozin (5–2,000 ng/mL), henagliflozin (5–2,000 ng/mL), and telmisartan (2–500 ng/mL). The peak area ratios of the target analytes to the IS were plotted against the plasma concentrations, which were weighted (1/x^2^), and the data were fit using linear least-squares regression. For validation, the accuracy and precision across all tested concentrations from these curves should not exceed 15%, except for the LLOQ, where a maximum of 20% variance is permissible.

#### Accuracy and precision.

Intra- and inter-day accuracy and precision were decided at low, medium, and high QC concentrations, as well as at the LLOQ over the course of three successive days. Precision was quantified by reckoned the relative standard deviation (RSD) from six replicates for each sample. Likewise, accuracy was measured by determining the relative error (RE) from these six repetitions. For validity, the RSD and RE values must lowered than ±15% for QC concentrations and ±20% at the LLOQ.

#### Matrix effect and extraction recovery.

Matrix effects were investigated by comparing the area under the peak areas of the analyte at three QC concentrations in post-extraction blank plasma with the peak areas of corresponding analytes in a solution composed solely of acetonitrile and water. Comparing the analyte areas derived from the extracted samples with those obtained from post-extracted samples spiked with the analytes to evaluated the extraction recovery at these three QC levels. These values were then normalized based on the IS peak area.

#### Stability.

The stability of the analyte was evaluated in various storage and disposition circumstances at low, medium, and high QC concentrations. Specifically, the conditions were as follows: room temperature (25 ℃) for a duration of 4 hours, autosampler temperature (15 ℃) for 6 hours, storage at −80 ℃ for 30 days, and after undergoing three freeze–thaw cycles ranging from −80 ℃ to 25 ℃.

### Quantitative real-time PCR (qRT-PCR) analysis

The experimental conditions for the qRT-PCR analysis were based on the articles already published by our research group [31].

### Statistical analysis

Statistical assessments of the pharmacokinetic parameters were conducted using DAS 2.2.1 software, developed by the Mathematical Pharmacology Professional Committee of China. Analysis was conducted in a non-compartmental model. The metrics such as the area under the concentration-time curve (AUC), maximum plasma concentration (C_max_), time to reach C_max_ (T_max_), elimination half-life (t_1/2_), plasma clearance volume per time unit (CL_z/F_), apparent volume of distribution (V_z_), and mean residence time (MRT) were expressed as mean ± standard deviation (SD). Comparisons corresponding test groups were conducted using SPSS 25.0 software (SPSS Inc., Chicago, IL, USA). Data sets adhering to normal distributions as determined by the Shapiro–Wilk test were analyzed using Student’s t-test, while data sets with non-normal distributions were examined using the Mann–Whitney U-test. A P-value of less than 0.05 (two-tailed) was deemed statistically significant for all tests.

## Results

### Method development

**Fig 2:**
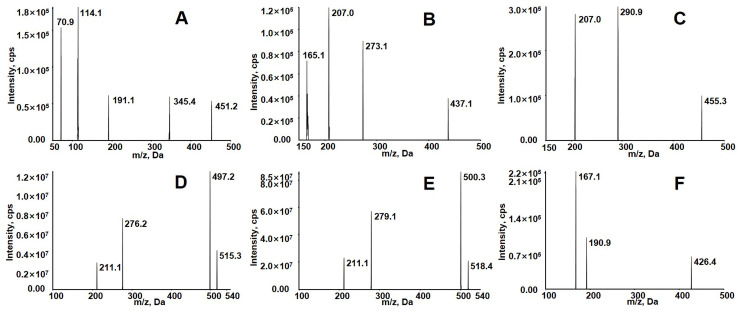
The mass spectra of empagliflozin(A), ertugliflozin (B), henagliflozin (C), telmisartan (D), telmisartan-d_3_(E), and dapagliflozin (F).

Compared with the UPLC-MS/MS method previously developed by our research group, Waters X Select HSS T3 column (2.1  × 100 mm, 2.5 µm) maintained at 40 ℃ achieved adequate separation of analytes. The gradient elution process featured an initial acetonitrile ratio in 50% and a flow rate in 0.3 mL/min, thereby achieving appropriate retention times for the simultaneous quantification of the four analytes in rat plasma. The choice of dapagliflozin as the common IS was influenced by its structural similarity to henagliflozin and ertugliflozin, as well as by published methodological studies on SGLT2 inhibitors [33]. Liquid-liquid extraction was preferred over protein precipitation as the sample preparation method to enhance column longevity and the reproducibility of high-throughput analyses. Ethyl acetate ether was used for this extraction, as it displayed superior efficiency compared to methyl tert-butyl ether. Mass spectrometric detection and quantification modes is both positive and MRM modes. Ion-monitoring transitions exhibiting the highest responses were recorded as m/z 451.2 → 70.9 for empagliflozin, 437.1 → 207.0 for ertugliflozin, 455.3 → 290.9 for henagliflozin, 515.3 → 497.2 for telmisartan, 518.4 → 279.1 for telmisartan-d_3_, and 426.4 → 167.1 for dapagliflozin(For the specific mass spectrum, please refer to Fig 2).

### Method validation

#### Selectivity.

**Fig 3:**
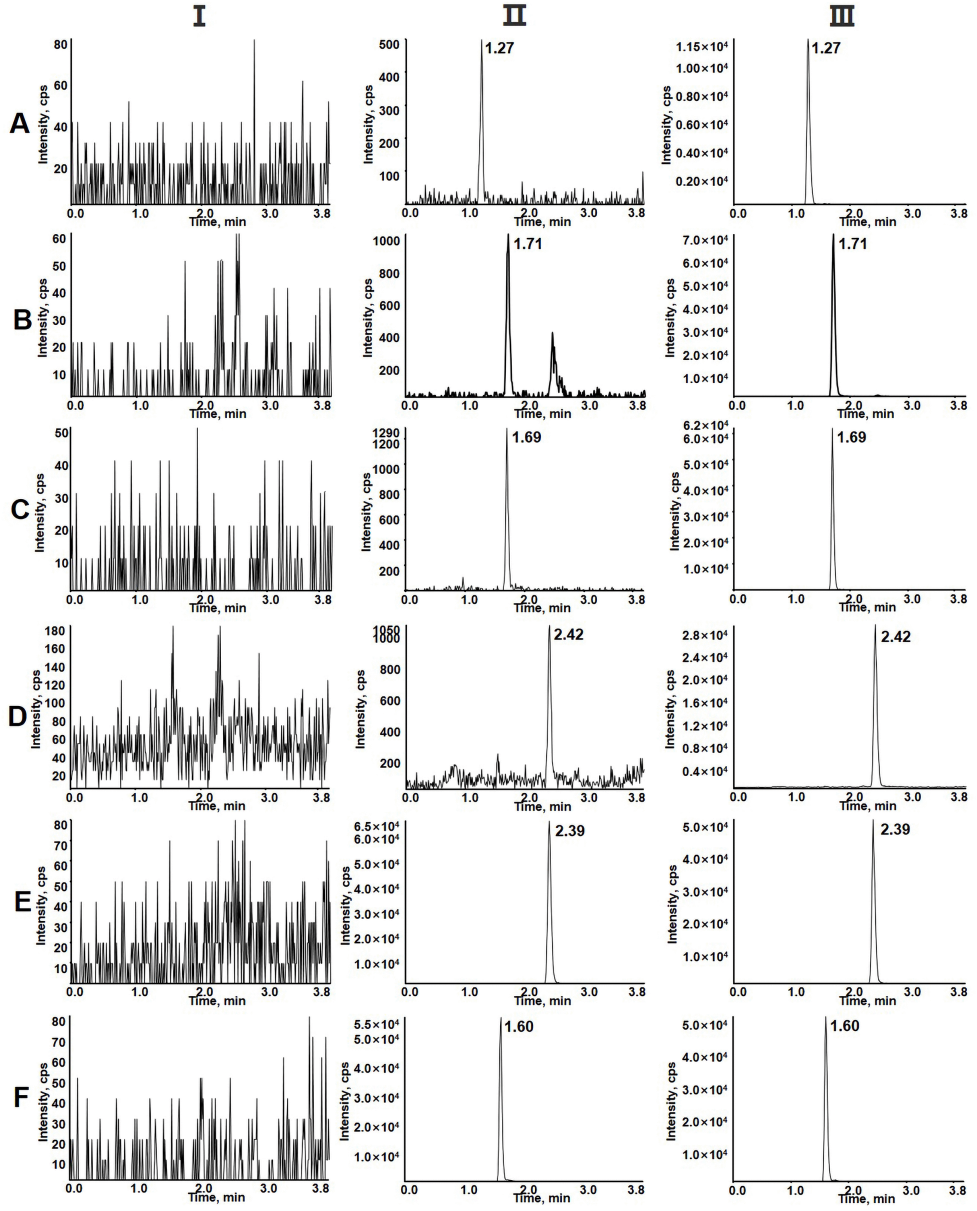
Representative chromatograms of empagliflozin (A), ertugliflozin (B), henagliflozin (C), telmisartan (D), telmisartan -d_3_(E), and dapagliflozin (F). I, a blank rat plasma sample; II, a blank rat plasma sample spiked with the working solution at the LLOQ level and IS; Ⅲ, a rat plasma sample after oral administration of 2.5 mg/kg empagliflozin, 1.5 mg/kg ertugliflozin,1 mg/kg henagliflozin, and 8 mg/kg telmisartan.

The method’s selectivity was assessed by comparing chromatograms from six distinct blank plasma samples drawn from various rats, plasma samples contained with the target analytes and the IS, as well as plasma samples from rats that had been administered the drugs under study. Fig 3 displays chromatograms from an unaltered blank sample, from contained blank samples at the LLOQ levels for each analyte along with the IS, and from drug-treated rats.

#### Calibration curve and LLOQ.

Calibration curves, plotting the ratio of peak area for each drug to that of the IS against the reciprocal of the square of the drug concentration (1/x^2^), demonstrated superb linearity with correlation coefficients exceeding 0.995. These curves were established over a concentration range of 5–2,000 ng/mL for empagliflozin, ertugliflozin and henagliflozin, and 2–500 ng/mL for telmisartan. These curves were established over a concentration range of 5–2,000 ng/mL for empagliflozin, ertugliflozin and henagliflozin, and 2–500 ng/mL for telmisartan. The equations for the calibration curves were as follows: Y =  0.000857x - 0.000894(r = 0.9997) for empagliflozin, Y =  0.00176x +  0.00378(r = 0.9996) for ertugliflozin, Y =  0.00292x - 0.00135(r = 0.9993) for henagliflozin, and Y =  0.00433x +  0.00499(r = 0.9996) for TEL. Here, Y represents the peak area ratio of each analyte to the IS, and x denotes the plasma concentration of the respective analyte. The established LLOQ values for empagliflozin, ertugliflozin, henagliflozin, and telmisartan were 5 ng/mL, 5 ng/mL, 5 ng/mL, and 2 ng/mL, respectively. Notably, neither the precision nor the accuracy exceeded by more than 20% from the standard LLOQ values.

#### Precision and accuracy.

The data for the precision and accuracy of QC samples at low-, medium-, and high-concentrations, as well as for the LLOQ samples, are exhibited in Table 1. The intra- and inter-day precision values did not exceed 8.9%, and the accuracy measurements spanned a range from −8.1% to 8.3% across all concentrations of the analytes in rat plasma. These findings suggest that the proposed analytical method is both repeatable and reproducible.

**Table 1:** Intra- and inter-day precision and accuracy of empagliflozin, ertugliflozin, henagliflozin, and telmisartan measurements in rat plasma(n = 6).

Analyte	Concentration (ng/ mL)	Intra-day (n = 6)			Inter-day (n = 18)		
		Mean ± SD	RSD (%)	RE (%)	Mean ± SD	RSD (%)	RE (%)
empagliflozin	5	5.18 ± 0.18	3.5	3.5	4.68 ± 0.42	8.9	−6.4
	10	10.80 ± 0.68	6.3	8.3	10.36 ± 0.79	7.6	3.6
	800	847.17 ± 45.67	5.4	5.9	829.28 ± 54.45	6.6	7.8
	1500	1540.00 ± 49.40	3.2	0.7	1564.44 ± 78.01	5.0	5.3
ertugliflozin	5	5.35 ± 0.39	7.3	−0.4	5.12 ± 0.43	8.4	2.4
	10	10.31 ± 0.38	3.7	3.1	10.30 ± 0.54	5.3	3.0
	800	838.83 ± 38.60	4.6	4.9	809.33 ± 54.40	6.7	−1.1
	1500	1606.67 ± 71.74	4.5	0.7	1564.44 ± 75.32	4.8	0.7
henagliflozin	5	4.60 ± 0.27	5.7	−8.1	4.62 ± 0.38	8.2	−7.6
	10	10.07 ± 0.45	4.5	0.6	10.15 ± 0.68	6.8	1.5
	800	830.17 ± 48.80	5.9	3.7	835.044 ± 53.76	6.4	4.4
	1500	1578.33 ± 58.45	3.7	−0.7	1521.67 ± 72.78	4.8	−0.7
telmisartan	2	1.87 ± 0.09	5.1	−6.7	1.85 ± 0.13	7.3	−7.4
	5	5.21 ± 0.29	5.5	4.1	4.99 ± 0.40	8.0	−0.1
	200	181.00 ± 6.32	3.5	−3.8	194.00 ± 13.02	6.7	2.0
	400	381.17 ± 17.88	4.7	−5.3	387.56 ± 29.32	7.6	−5.3

#### Matrix effects and extraction recovery.

Table 2 exhibited the matrix effect and extraction recovery results. The matrix effects for empagliflozin, ertugliflozin, henagliflozin, and telmisartan were within the range of 95.9–100.4%, 96–104.2%, 99.2–102.8%, and 98.8–106.9%, respectively. These values indicate an absence of significant matrix interference in rat plasma. Furthermore, the recovery rates, which were normalized by the IS peak area, ranged from 80.3% to 93.9%. The RSD was under 9.2% for all analytes, manifest that the liquid–liquid extraction process employed is efficient, reliable, and reproducible.

**Table 2:** Matrix effect and extraction recovery of empagliflozin, ertugliflozin, henagliflozin, and telmisartan from rat plasma (n  =  6).

Analyte	Concentration (ng/ mL)	Matrix effect		Extraction recovery	
		Mean ± SD (%)	RSD (%)	Mean ± SD (%)	RSD (%)
empagliflozin	10	95.9 ± 3.98	4.1	80.3 ± 5.48	6.8
	800	99.5 ± 3.56	3.6	91.2 ± 3.68	4.0
	1500	100.4 ± 5.05	5.0	90.5 ± 8.13	9.0
ertugliflozin	10	98.1 ± 3.69	3.8	90.1 ± 8.25	9.2
	800	96.0 ± 4.39	4.6	90.9 ± 5.74	6.3
	1500	104.2 ± 3.38	3.2	92.1 ± 6.71	7.3
henagliflozin	10	99.2 ± 4.64	4.7	88.9 ± 4.65	5.2
	800	102.8 ± 5.48	5.3	90.2 ± 5.01	5.5
	1500	102.7 ± 4.75	4.6	93.9 ± 2.58	2.7
telmisartan	5	106.9 ± 5.55	5.2	87.6 ± 4.55	5.2
	200	99.5 ± 4.41	4.4	93.4 ± 2.60	2.8
	400	98.8 ± 5.08	5.1	90.4 ± 6.01	6.7

#### Stability.

Stability assessments of the target analytes in QC samples were proceed under various storage and handling conditions: indoor temperature, 15 °C in the autosampler, −80 ℃, and after passing three freeze–thaw cycles. The outcomes are exhibited in Table 3. For all experimental circumstances, both the RE and RSD values kept below 9.9% and 8.1%, respectively. This implies that the analytes maintain excellent stability when treated to the specified preparation, storage, and analytical orders.

**Table 3:** Stability of empagliflozin, ertugliflozin, henagliflozin, and telmisartan in rat plasma (n  =  6).

Analyte	Conditions	Concentration (ng/mL)	Mean ± SD (ng/ml)	Precision (RSD%)	Accuracy (RE%)
empagliflozin	Autosampler (15 ºC) for 6 h	10	10.48 ± 0.64	6.1	4.8
		800	879.33 ± 46.19	5.3	9.9
		1500	1606.67 ± 88.69	5.5	7.1
	Room temperature for 4 h	10	10.25 ± 0.61	6.0	2.5
		800	841.00 ± 47.58	5.7	5.1
		1500	1556.67 ± 42.27	2.7	3.8
	−80 ℃ for 30 days	10	10.22 ± 0.39	3.7	2.2
		800	800.33 ± 64.85	8.1	0.1
		1500	1513.33 ± 73.67	4.9	0.9
	Three freeze–thaw cycles (−80 ℃ to 25 ºC)	10	9.34 ± 0.34	3.6	−6.6
		800	827.33 ± 23.76	2.9	3.4
		1500	1591.67 ± 43.09	2.7	6.1
ertugliflozin	Autosampler (15 ºC) for 6 h	10	10.76 ± 0.62	5.7	7.6
		800	851.33 ± 26.52	3.1	6.4
		1500	1596.67 ± 61.21	3.8	6.4
	Room temperature for 4 h	10	10.32 ± 0.34	3.2	3.2
		800	811.5 ± 30.51	3.8	1.4
		1500	1506.67 ± 44.57	3.0	0.4
	−80 ℃ for 30 days	10	10.25 ± 0.69	6.7	2.5
		800	790.50 ± 36.40	4.6	−1.1
		1500	1515.00 ± 50.50	3.3	1.0
	Three freeze–thaw cycles (−80 ℃ to 25 ºC)	10	9.63 ± 0.64	6.7	−3.7
		800	790.67 ± 42.87	5.4	−1.1
		1500	1528.33 ± 82.08	5.4	1.9
henagliflozin	Autosampler (15 ºC) for 6 h	10	10.53 ± 0.71	6.8	5.3
		800	860.50 ± 40.55	4.7	7.6
		1500	1540.00 ± 56.21	3.7	2.7
	Room temperature for 4 h	10	9.74 ± 0.52	5.3	−2.6
		800	802.00 ± 39.39	4.9	0.2
		1500	1475.00 ± 39.37	2.7	−1.6
	−80 ℃ for 30 days	10	10.06 ± 0.33	3.2	0.6
		800	767.00 ± 32.77	4.3	−4.1
		1500	1436.67 ± 36.70	2.6	−4.2
	Three freeze–thaw cycles (−80 ℃ to 25 ºC)	10	9.97 ± 0.58	5.8	−0.2
		800	847.17 ± 45.67	5.4	5.9
		1500	1621.67 ± 73.87	4.6	8.1
telmisartan	Autosampler (15 ºC) for 6 h	5	4.64 ± 0.32	7.0	−7.1
		200	192.33 ± 6.06	3.1	−3.8
		400	386.33 ± 16.95	4.4	−3.4
	Room temperature for 4 h	5	5.21 ± 0.24	4.5	4.1
		200	187.17 ± 5.27	2.8	−6.4
		400	365.67 ± 26.06	7.1	−8.6
	−80 ℃ for 30 days	5	4.94 ± 0.38	7.8	−1.1
		200	202.00 ± 5.37	2.7	1.0
		400	391.33 ± 26.46	6.8	−2.2
	Three freeze–thaw cycles (−80 ℃ to 25 ºC)	5	4.89 ± 0.29	6.0	−2.2
		200	188.67 ± 15.24	8.1	−5.7
		400	393.83 ± 28.38	7.2	−1.5

### Pharmacokinetic interactions

The influence of empagliflozin, ertugliflozin and henagliflozin on the pharmacokinetics of telmisartan

**Fig 4:**
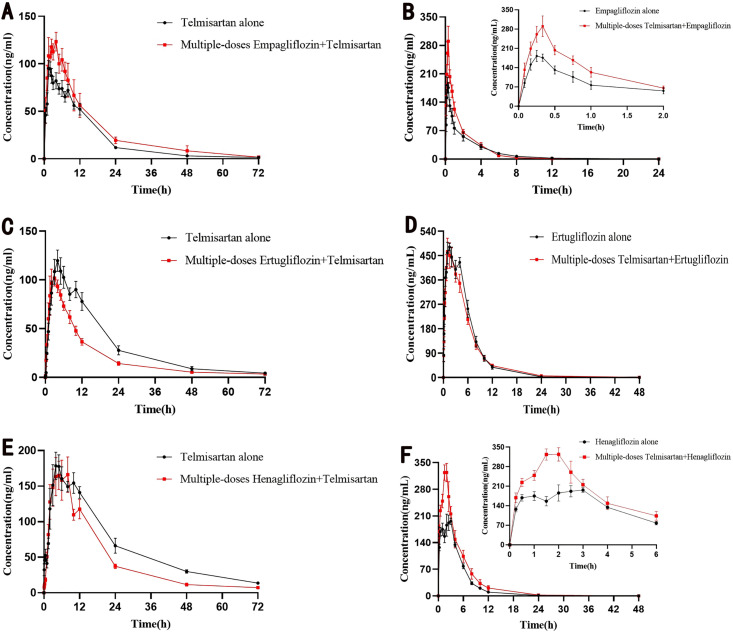
The mean plasma concentration–time profiles for empagliflozin, ertugliflozin henagliflozin, telmisartan following oral administration either alone or in conjunction with multiple-doses corresponding drug formulation.

The mean plasma concentration-time curves of all drug combinations after oral administration are all presented in Fig 4. The mean concentration–time profiles for telmisartan administered alone and in combination with multiple doses of empagliflozin are shown in Fig 4A. The main pharmacokinetic parameters of telmisartan obtained using non-compartmental methods are summarized in Table 4. The results indicate that co-administration with empagliflozin does not lead to significant alterations in the pharmacokinetic parameters of telmisartan.

**Table 4:** Pharmacokinetic parameters of empagliflozin, ertugliflozin, henagliflozin and telmisartan in rat plasma following oral administration of single dose and combined doses(n  =  6).

Parameters (Unit)	telmisartan (8 mg/kg)		empagliflozin (2.5 mg/kg)	
	Alone	After empagliflozin	Alone	After telmisartan
AUC_0 − t_ (μg/L*h)	1870.45 ± 304.58	1647.55 ± 218.40	336.31 ± 7.89	434.37 ± 52.41 *
AUC_0−∞_ (μg/L*h)	1882.69 ± 308.01	1669.06 ± 234.77	353.21 ± 44.23	439.97 ± 49.86*
C_max_ (μg/L)	119.57 ± 15.73	134.50 ± 22.71	188.67 ± 47.22	297.00 ± 80.22 *
T_max_ (h)	1.92 ± 0.38	2.58 ± 0.86	0.29 ± 0.07	0.30 ± 0.07
t_1/2z_ (h)	9.27 ± 3.71	10.43 ± 2.780	1.67 ± 0.4	1.16 ± 0.42
CL_z_ (L/h/kg)	4.35 ± 0.71	4.87 ± 0.67	7.18 ± 0.95	5.74 ± 0.60 *
V_z_ (L/kg)	58.31 ± 22.72	71.45 ± 12.42	17.44 ± 5.71	9.72 ± 4.01 *
MRT_0 − t_ (h)	12.50 ± 1.62	14.09 ± 3.75	2.18 ± 0.52	1.74 ± 0.32
MRT_0−∞_ (h)	12.98 ± 1.96	14.95 ± 4.86	2.59 ± 0.70	1.85 ± 0.40 *
Ka (1/h)	0.80 ± 0.16	1.23 ± 0.66	8.37 ± 2.13	7.07 ± 1.22
Ke (1/h)	0.09 ± 0.02	0.07 ± 0.01	0.46 ± 0.18	0.66 ± 0.23
Parameters (Unit)	telmisartan (8 mg/kg)		ertugliflozin (1.5 mg/kg)	
	Alone	After ertugliflozin	Alone	After telmisartan
AUC_0 − t_ (μg/L*h)	2769.66 ± 887.87	1530.86 ± 235.03*	3226.50 ± 480.06	3018.55 ± 537.29
AUC_0−∞_ (μg/L*h)	2850.97 ± 892.93	1645.61 ± 318.08*	3237.02 ± 488.72	3041.37 ± 542.53
C_max_ (μg/L)	145.17 ± 47.70	118.88 ± 29.55	522.33 ± 52.82	506.00 ± 104.15
T_max_ (h)	4.67 ± 1.03	2.17 ± 0.68**	1.50 ± 0.45	1.67 ± 0.52
t_1/2z_ (h)	13.84 ± 4.08	18.93 ± 8.97	2.68 ± 0.56	3.25 ± 0.75
CL_z_ (L/h/kg)	3.04 ± 0.95	5.00 ± 0.89**	0.47 ± 0.08	0.51 ± 0.09
V_z_ (L/kg)	61.47 ± 26.85	131.99 ± 51.25*	1.79 ± 0.28	2.36 ± 0.63
MRT_0 − t_ (h)	16.99 ± 2.29	16.81 ± 3.71	4.65 ± 0.54	4.94 ± 0.45
MRT_0−∞_ (h)	19.28 ± 3.90	22.57 ± 10.44	4.72 ± 0.60	5.12 ± 0.61
Ka (1/h)	0.33 ± 0.07	0.86 ± 0.14 *	0.25 ± 0.05	0.57 ± 0.12 *
Ke (1/h)	0.05 ± 0.02	0.04 ± 0.02	0.27 ± 0.06	0.22 ± 0.06
Parameters (Unit)	telmisartan (8 mg/kg)		henagliflozin (1 mg/kg)	
	Alone	After henagliflozin	Alone	After telmisartan
AUC_0 − t_ (μg/L*h)	4144.24 ± 1101.51	3009.01 ± 398.45*	1140.54 ± 188.97	1640.91 ± 490.12*
AUC_0−∞_ (μg/L*h)	4621.46 ± 1082.10	3069.23 ± 390.33**	1145.52 ± 190.90	1651.04 ± 492.09*
C_max_ (μg/L)	160.33 ± 24.57	148.50 ± 23.76	217.33 ± 40.52	356.83 ± 59.55**
T_max_ (h)	3.67 ± 1.21	4.33 ± 1.37	2.33 ± 0.82	1.67 ± 0.52
t_1/2z_ (h)	23.33 ± 7.27	11.79 ± 2.96*	2.99 ± 0.92	3.47 ± 0.63
CL_z_ (L/h/kg)	1.84 ± 0.59	2.65 ± 0.38*	0.89 ± 0.14	0.64 ± 0.16 *
V_z_ (L/kg)	61.94 ± 23.48	45.04 ± 12.31	3.87 ± 1.49	3.23 ± 1.01
MRT_0 − t_ (h)	21.64 ± 0.62	18.84 ± 2.91	4.22 ± 0.21	4.38 ± 0.53
MRT_0−∞_ (h)	31.04 ± 5.40	20.28 ± 3.94**	4.33 ± 0.22	4.54 ± 0.50
Ka (1/h)	0.44 ± 0.06	0.37 ± 0.12	2.39 ± 0.63	1.39 ± 0.37
Ke (1/h)	0.03 ± 0.01	0.06 ± 0.01 **	0.25 ± 0.08	0.21 ± 0.04

*P < 0.05, **P < 0.01, compared with control group, manifesting statistically remarkable difference. The main pharmacokinetic parameters are shown as the mean ± standard deviation.

In a similar experimental setup, the mean plasma concentration–time profiles for telmisartan following oral administration either alone or in conjunction with multiple doses of ertugliflozin are depicted in Fig 4C. The core pharmacokinetic parameters are detailed in Table 4. Upon administration of ertugliflozin, the AUC_0-t_ and AUC_0-∞_ of telmisartan were significantly reduced by 44.73%(P = 0.018) and 42.28%(P = 0.020), respectively. Concurrently, CL_z/F_, Ka and V_z_ saw significant increments of 64.50%(P = 0.004), 159.82%(P = 0.011) and 114.74%(P = 0.019), respectively. Moreover, the T_max_ of telmisartan was reduced from 4.67 to 2.17 hours(P = 0.003). However, ertugliflozin did not significantly impact the C_max_, t_1/2_, Ke, MRT_0-t_, and MRT_0-∞_ of telmisartan.

Lastly, the mean plasma concentration–time profiles for telmisartan following oral administration either alone or in conjunction with multiple doses of henagliflozin are displayed in Fig 4E, with the primary pharmacokinetic parameters summarized in Table 4. Following henagliflozin administration, the AUC_0-t_, AUC_0-∞_, t_1/2_, and MRT_0-∞_ of telmisartan decreased by 27.39%(P = 0.039), 33.59%(P = 0.008), 49.47%(P = 0.010) and 34.68%(P = 0.003), respectively. Additionally, the CL_z/F_ and Ke showed an increase of 43.65%(P = 0.037) and 90.63%(P = 0.002), respectively. On the other ha /nd, henagliflozin had no discernible impact on the T_max_, MRT_0−t_, V_z_, Ka, and C_max_ of telmisartan.

The influence of telmisartan on the pharmacokinetics of empagliflozin, ertugliflozin or henagliflozin

The mean plasma concentration–time curves of empagliflozin when administered alone and when coadministered with telmisartan are presented in Fig 4B, and the main pharmacokinetic parameters of empagliflozin are displayed in Table 4. When coadministered with telmisartan, the AUC_0-t_, AUC_0-∞_, and C_max_ of empagliflozin increased by 29.16%(P = 0.004), 24.56%(P = 0.010) and 57.42%(P = 0.021), respectively. In contrast, the CL_z/F_ decreased by 20.05%(P = 0.010), and the V_z_ was reduced by 44.28%(P = 0.022). Additionally, the MRT_0−∞_ was 28.73%(P = 0.047) shorter. However, telmisartan had no significant impact on the T_max_, t_1/2_, Ka, Ke, and MRT_0−t_ of empagliflozin.

The arithmetic means of the plasma concentrations of ertugliflozin after oral administration alone or following multiple doses of telmisartan are presented in Fig 4D. The main pharmacokinetic parameters are listed in Table 4. Comparative analysis reveals that co-administration with telmisartan only increased the Ka of ertugliflozin by 126.09%, Other parts did not bring about significant changes in the pharmacokinetic parameters of ertugliflozin.

Lastly, the mean plasma concentration–time profiles for henagliflozin following oral administration either alone or in conjunction with multiple doses of telmisartan are depicted in Fig 4F. The relevant pharmacokinetic parameters are summarized in Table 4. In rats treated with multiple doses of telmisartan, the AUC_0−t_, AUC_0−∞_, and C_max_ of henagliflozin increased by 43.88%(P = 0.010), 44.13%(P = 0.010), and 64.19%(P = 0.001), respectively. Additionally, the CL_z/F_ was reduced by 28.44%(P = 0.010). On the other hand, telmisartan had no noticeable influence on the T_max_, t_1/2_, V_z_, Ka, Ke, MRT_0−t_, and MRT_0−∞_ of henagliflozin.

### messenger RNA (mRNA) expression in rat liver, intestines and kidney

**Fig 5:**
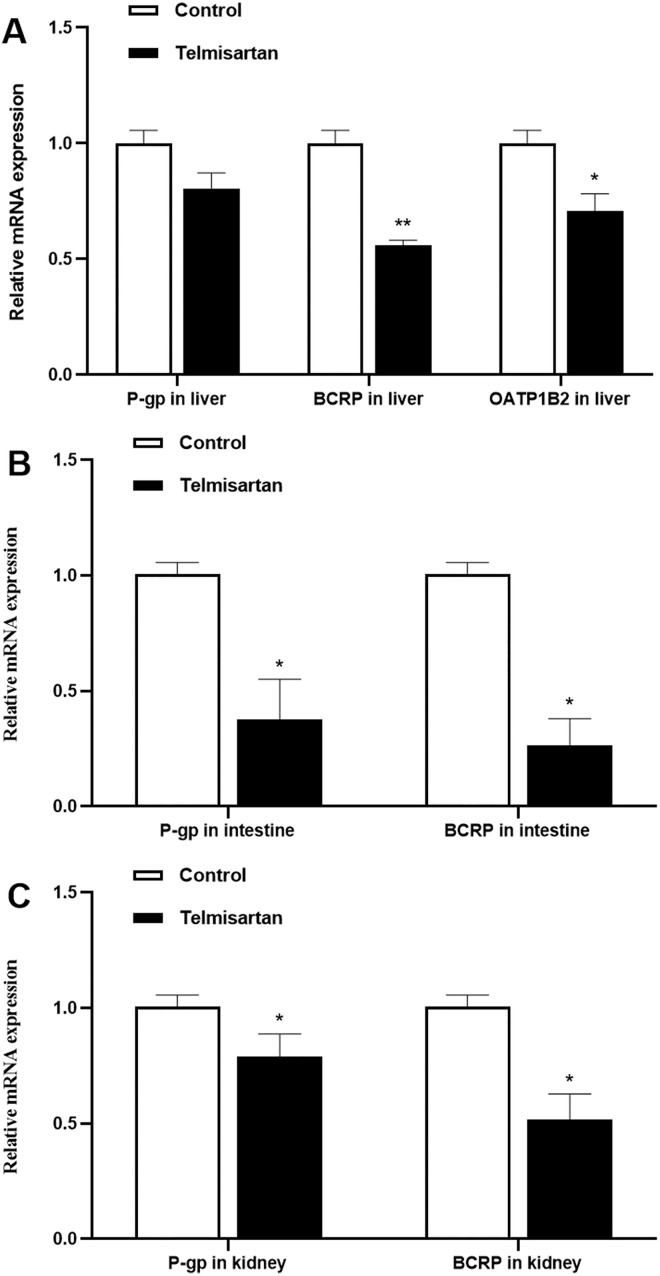
Relative mRNA expression of P-gp and BCRP in the liver, intestines, and kidney and OATP1B2 in liver. **(A)** Effect of multiple-doses telmisartan administration on mRNA expression of P-gp, BCRP and OATP1B2 in the liver; **(B)** Effect of multiple-doses telmisartan administration on mRNA expression of P-gp and BCRP in the intestines; **(C)** Effect of multiple-doses telmisartan administration on mRNA expression of P-gp and BCRP in the kidney. * P <  0.05, * * P <  0.01.

The relative mRNA expression levels in the liver, intestine and kidney of the rats are shown in Fig 5. Because OATP1B1/1B3 is a pseudogene, there is no expression in rats, and its function in rats is replaced by OAPT1B2. Therefore, we assess the mRNA expression of OAPT1B2 in rat to evaluate its role in the pharmacokinetic interactions. After the administration of telmisartan for seven consecutive days, the mRNA expression of BCRP and OATP1B2 in the liver lowered by 44.1%(P = 0.003) and 29.1%(P = 0.016), respectively. However, no significant alteration in the mRNA expression of P-gp was noticed in the liver (Fig 5A). Additionally, after multiple doses of telmisartan, mRNA expression of P-gp and BCRP in the intestines was reduced by 62.4%(P = 0.023) and 73.4%(P = 0.037), respectively (Fig 5B). Interestingly, after multiple doses of telmisartan, mRNA expression of P-gp and BCRP in the kidney was reduced by 21.1%(P = 0.048) and 48.2%(P = 0.037), respectively (Fig 5C).

## Discussion

SGLT2 inhibitors have emerged as a novel remedy choice for diabetes. Individuals with diabetes often have other chronic comorbidities, such as cardiovascular, renal, or metabolic disorders, necessitating additional medication. Moreover, many drugs used in these therapeutic regimes are metabolized and cleared by the cytochrome P450 (CYP450) enzymes, UGTs, and various transporters. Some are also substrates or inhibitors of these drug-metabolizing enzymes and transporters, thereby increasing the risk of DDIs. DDIs may alter pharmacokinetic and pharmacodynamic properties, either reducing treatment efficacy or generating adverse drug reactions. In the standard remedy strategy of cotreatment with antidiabetic and antihypertensive medicine for T2DM with hypertension. ARBs like telmisartan and SGLT2 inhibitors like empagliflozin, ertugliflozin, and henagliflozin are the substrates of P-gp and BCRP, telmisartan also is the inhibitor of P-gp and BCRP. Given these properties, assessing the impact of pharmacokinetics, curative effect, and adverse effects between the two types of medication is essential.

### Effects of empagliflozin, ertugliflozin and henagliflozin on telmisartan pharmacokinetics

When coadministration with empagliflozin, ertugliflozin or henagliflozin, empagliflozin had no significant effect on the exposure of telmisartan, while ertugliflozin and henagliflozin were reduced the exposure of telmisartan. According to the in vitro evaluation reports of ertugliflozin and henagliflozin respectively by Merck Sharp & Dohme B.V. and Jiangsu Hengrui Pharmaceutical Co., LTD., neither of the two drugs were likely to have inhibitory effects on the transporters P-gp and OATP1B1/1B3. Therefore, it is unlikely to have an impact on telmisartan from the aspect of transporters.

Telmisartan was rarely metabolized by UGT1A3. However, previous studies had shown that overexpression of UGT1A3 can to some extent affect the AUC of telmisartan [21,34]. Ertugliflozin had no significant effect on the t_1/2_ of telmisartan, while henagliflozin significantly shortened the t_1/2_ of telmisartan. The most likely possibility was henagliflozin affected the metabolism of telmisartan, but there was no detailed study yet showing the relationship between henagliflozin and UGT1A3, and further research was worth it.

In addition, the plasma protein binding rates of ertugliflozin and henagliflozin were both higher than those of empagliflozin, indicated that the two drugs have a higher plasma protein binding capacity. Moreover, telmisartan itself has an extremely high plasma protein binding rate. It is highly likely that when the two drugs are used in combination, they will compete for the same protein binding sites. This competition would increase the free drug concentration of telmisartan in the plasma, leading to enhanced tissue distribution and accelerated clearance. These findings align with those of another study [26]. On the other hand, from the changes in the Ka values of the drugs, we can see that after multiple administrations of ertugliflozin, the absorption of telmisartan was accelerated, while no such change occurred after multiple administrations of henagliflozin. We can speculate that ertugliflozin may have the same protein binding sites as telmisartan, but further experiments were needed to further verify this possibility. Another potential mechanism could involve is ertugliflozin and henagliflozin had the similar structure like dapagliflozin [35]. They might affect the gut microbiota, thereby alter the absorption and tissue distribution of telmisartan and consequently leading to changes in pharmacokinetic parameters. This situation requires further research to confirm.

Moving forward, our investigations will focus on determining the free drug concentrations in plasma, identifying drug binding sites on plasma proteins using molecular docking, and delving deeper into the exact mechanism.

### Effects of telmisartan on empagliflozin, ertugliflozin or henagliflozin pharmacokinetics

An increase in AUC and C_max_ and decrease in CL_z/F_ and V_z_ of empagliflozin were observed in rats for coadministration with telmisartan. First, in vitro research shows that empagliflozin is a substrate of OATP1B1/1B3 [15]. Thus, telmisartan might inhibit the OATP1B1/1B3-mediated hepatic uptake of empagliflozin, resulting in increased free drug concentration. This finding was consistent with the known inhibitory effects of telmisartan on Cilostazol hepatic uptake [36]. In addition, both telmisartan and empagliflozin are substrates of P-gp and BCRP, and telmisartan also can Inhibit these. Therefore, telmisartan could further heighten empagliflozin exposure by inhibiting these efflux transporters. Furthermore, telmisartan is known to influence certain enzymes, including UGT1A9, which is a target gene of PPAR-α/γ, while empagliflozin was metabolized through multiple UGT enzymes [37]. Therefore, telmisartan was unlikely to metabolically affect the exposure of empagliflozin in the body. Remarkably, our study found that telmisartan notably downregulated the mRNA expression levels of P-gp, BCRP, and OATP1B2 in the rat liver, intestines, and kidney upon multiple oral doses. These results suggest that inhibition of excretion pathways might contribute to the observed increase in empagliflozin exposure following multiple doses of telmisartan.

When co-administered with multiple doses of telmisartan, the pharmacokinetic parameters of ertugliflozin did not exhibit similar alterations to those observed with empagliflozin. Firstly, both telmisartan and ertugliflozin are drugs with high protein-binding affinity, and they may compete for identical protein-binding sites. This competition could elevate the concentration of free ertugliflozin in plasma, subsequently leading to enhanced tissue distribution and more rapid drug clearance. This hypothesis is supported by the noticeable increase in the V_z_ and Ka of telmisartan following co-administration with multiple doses of ertugliflozin. Similarly, after multiple administrations of telmisartan, the Ka value of empagliflozin also significantly increased, indicated that there might be a higher concentration of empagliflozin in the plasma. Ertugliflozin is also a substrate of P-gp [11], and telmisartan may increase in vivo exposure to ertugliflozin by inhibiting P-gp. However, since UGT1A9 is the main enzyme system for the metabolism of ertugliflozin [14], the induction effect of telmisartan on the enzyme system may accelerate the metabolism of ertugliflozin, leading to a reduction in its exposure in the body. The combined effect of multiple factors may explain the experimental results of this part.

Compared with empagliflozin, henagliflozin had a more similar drug structure to ertugliflozin. However, when it is co-administered with telmisartan, it exhibits pharmacokinetic characteristics more similar to those of empagliflozin. Telmisartan increased the in vivo exposure of henagliflozin and reduced its clearance rate, but has no significant effect on the V_z_ of henagliflozin. This situation may be related to the subtle structural differences among the three SGLT2 inhibitor drugs. Similarly, telmisartan was unlikely to have a significant impact on the metabolism of henagliflozin. Since henagliflozin was also a substrate of P-gp and BCRP, telmisartan can also inhibit the increase in vivo exposure to henagliflozin caused by these transporters.

Coadministration of these drugs alters the pharmacokinetic profile of each drug due to DDIs, potentially leading to complex consequences, including heightened risk of severe adverse effects or reduced therapeutic efficacy. Given the alterations in the AUC, C_max_, or CL_z/F_ of empagliflozin and henagliflozin when taken with telmisartan, doctors should be attention the possibility of this DDI and monitor hemoglobin A1c levels when two drugs are co-administered.However, the risks associated with this interaction also required more clinical evidence to verify.

## Conclusion

We made some improvements to the previously published method to make it more suitable for this experiment and then verified it. Subsequently, we employed it to assess the impact of empagliflozin, ertugliflozin, and henagliflozin on the pharmacokinetics of telmisartan, and vice versa. We discovered that the pharmacokinetic interactions between the three SGLT2 inhibitors and telmisartan are mainly related to P-gp, BCRP and MRP2. We also observed some notable phenomena: Co-administration with multiple doses of telmisartan resulted in differential effects on the pharmacokinetics of empagliflozin, ertugliflozin, and henagliflozin. Specifically, co-administration with telmisartan led to elevated systemic exposure to empagliflozin and henagliflozin, but left the exposure to ertugliflozin unaffected in vivo. These alterations may be explained by the discrepancies in the structural features of these drugs. It is imperative to conduct further investigations to elucidate how fine structural optimizations within the same drug class can impact DDIs. Increased understanding of the drug-drug interactions between telmisartan and empagliflozin, ertugliflozin, or henagliflozin can aid in the precise dosage adjustment, mitigating adverse reactions, and ensuring effective treatment for patients. Nevertheless, it is essential to emphasize that all our findings warrant further validation through clinical trials involving patient populations, including comprehensive evaluations of treatment responses.
